# Supplementary material for: Factors Associated with the Patient’s Decision to Avoid Healthcare during the COVID-19 Pandemic
Source: Int J Environ Res Public Health. 2021 Dec 15;18(24):13239. doi: 10.3390/ijerph182413239 (PMC8701299; doi:10.3390/ijerph182413239)
Supplement: Supplementary file 1 [file ijerph-18-13239-s001.zip › ijerph-1475165-supplementary.pdf]

Table S1. Prevalence ratios for healthcare avoidance, adjusted for sex, age group, region, education, health status and pandemic period.

	Crude		Adjusted	
	PR	95% CI	PR	95% CI
**Sex** (ref. Male)	**1.26**	**(1.19; 1.34)**	**1.27**	**(1.20; 1.35)**
**Age** (ref. 26-65)				
18-25	**0.86**	**(0.76; 0.98)**	**0.86**	**(0.75; 0.98)**
>65	0.97	(0.91; 1.04)	0.98	(0.92; 1.05)
**Region** (ref. Lisbon and Tagus Valey)				
North	**0.93**	**(0.87; 0.98)**	**0.92**	**(0.87; 0.98)**
Center	**0.93**	**(0.86; 0.99)**	**0.92**	**(0.86; 0.98)**
Alentejo	1.06	(0.95; 1.18)	1.07	(0.96; 1.19)
Algarve	1.06	(0.95; 1.18)	1.06	(0.95; 1.18)
Azores	**0.67**	**(0.50; 0.90)**	**0.66**	**(0.49; 0.89)**
Madeira	0.71	(0.49; 1.03)	0.72	(0.50; 1.03)
**Education** (ref. University)				
Basic/None	0.95	(0.86; 1.05)	0.95	(0.86; 1.05)
Secondary	0.96	(0.90; 1.01)	0.95	(0.90; 1.00)
**Occupation** (ref. Worker)				
Student	0.93	(0.82; 1.06)	1.06	(0.88; 1.26)
Retired	1.02	(0.96; 1.09)	1.08	(0.98; 1.19)
Unemployed	1.01	(0.91; 1.13)	1.02	(0.92; 1.14)
Other	1.08	(0.99; 1.18)	1.06	(0.97; 1.17)
**Confidence in the capacity of health services to respond to COVID-19** (ref. High)				
Low	**1.21**	**(1.15; 1.27)**	**1.19**	**(1.13; 1.25)**
**Confidence in the capacity of health services to respond to non-COVID-19** (ref. High)				
Low	**1.26**	**(1.20; 1.32)**	**1.24**	**(1.18; 1.30)**
**Monthly household income** (ref. >2501€)				
<650€	0.99	(0.89; 1.11)	0.98	(0.87; 1.11)
651-1000€	1.08	(1.00; 1.17)	1.07	(0.98; 1.16)
1001-1500€	1.06	(0.98; 1.14)	1.02	(0.95; 1.10)
1501-2000€	1.02	(0.95; 1.11)	1.02	(0.94; 1.10)
2001-2500€	1.07	(0.99; 1.16)	1.04	(0.97; 1.13)
**Lost of income due to the pandemic** (ref. No)	**1.09**	**(1.04; 1.15)**	**1.10**	**(1.04; 1.15)**
**Perception of the health status** (ref. Very good/Good)				
Reasonable	**1.26**	**(1.20; 1.32)**	**1.25**	**(1.19; 1.31)**
Bad/Very bad	**1.37**	**(1.23; 1.52)**	**1.38**	**(1.23; 1.54)**
**Number of comorbidities** (ref. 0)				
1	**1.12**	**(1.06; 1.18)**	**1.06**	**(1.01; 1.12)**
≥2	**1.11**	**(1.04; 1.18)**	1.02	(0.96; 1.10)
**Frequency of agitation, sadness or anxiety due to the physical distance measures** (ref. Never)				
Some days	**1.34**	**(1.24; 1.43)**	**1.24**	**(1.15; 1.33)**

	Crude		Adjusted	
	PR	95% CI	PR	95% CI
Almost every day	**1.71**	**(1.58; 1.85)**	**1.54**	**(1.42; 1.67)**
Every day	**1.77**	**(1.61; 1.94)**	**1.57**	**(1.42; 1.72)**
**Pandemic period** (ref. P6)				
P2	**1.18**	**(1.09; 1.27)**	**1.20**	**(1.11; 1.30)**
P3	**1.09**	**(1.01; 1.18)**	**1.13**	**(1.04; 1.22)**
P4	**1.23**	**(1.16; 1.31)**	**1.25**	**(1.18; 1.33)**
P5	**1.09**	**(1.02; 1.16)**	**1.08**	**(1.01; 1.16)**
**Self-Perceived Risk to get COVID-19 Infection** (ref. High)				
Moderate	0.94	(0.88; 1.01)	0.98	(0.91; 1.05)
Low/No risk	**0.81**	**(0.76; 0.88)**	**0.88**	**(0.82; 0.95)**
Unsure	0.91	(0.82; 1.01)	0.94	(0.84; 1.04)
**Self-Perceived Risk to Develop Severe Disease Following COVID-19 Infection** (ref. High)				
Moderate	**0.92**	**(0.87; 0.98)**	0.98	(0.92; 1.04)
Low/No risk	**0.74**	**(0.69; 0.79)**	**0.82**	**(0.77; 0.88)**
Unsure	**0.86**	**(0.80; 0.93)**	**0.91**	**(0.84; 0.99)**
**Self-perceived risk to get infected in a health institution** (ref. High)				
Moderate	**0.75**	**(0.70; 0.79)**	**0.74**	**(0.70; 0.79)**
Low/No risk	**0.44**	**(0.41; 0.48)**	**0.44**	**(0.40; 0.48)**
Unsure	**0.73**	**(0.63; 0.86)**	**0.73**	**(0.63; 0.85)**
**Perception of the adequacy of the measures implemented by the Government** (ref. Positive)				
Negative	**1.06**	**(1.01; 1.11)**	**1.07**	**(1.02; 1.12)**
**View on the information provided by the health authorities** (ref. Clear and understandable)				
Unclear and confusing	**1.12**	**(1.03; 1.21)**	**1.11**	**(1.03; 1.21)**
Inconsistent and contradictory	**0.90**	**(0.82; 0.98)**	0.92	(0.83; 1.01)

PR: prevalence ratio, CI: confidence interval.
